# Applying an ELSI lens to real-world data and novel genomic insights for personalized mental healthcare

**DOI:** 10.3389/fgene.2024.1444084

**Published:** 2024-08-14

**Authors:** Rachele M. Hendricks-Sturrup, Sandra E. Yankah, Christine Y. Lu

**Affiliations:** ^1^ Duke-Robert J. Margolis, MD, Institute for Health Policy, Washington, DC, United States; ^2^ Department of Population Medicine, Harvard Pilgrim Health Care Institute and Harvard Medical School, Boston, MA, United States; ^3^ Kolling Institute, Faculty of Medicine and Health, The University of Sydney and The Northern Sydney Local Health District, Sydney, NSW, Australia; ^4^ School of Pharmacy, Faculty of Medicine and Health, The University of Sydney, Sydney, NSW, Australia

**Keywords:** genetics, environment, personalized medicine, psychiatry, social determinants of health, real-world data, real-world evidence, genomics

## Abstract

Improving the understanding of the complex relationship between genetic predispositions, environmental influences, and sociocultural factors in the development and progression of mental illness is crucial for optimizing treatment efficacy and addressing longstanding health disparities. This paper discusses the ethical, legal, and social implications (ELSI) of recent advancements in biomedical research, particularly in genome-wide association studies (GWAS), phenome-wide association studies (PheWAS), and genome-wide environment interaction studies (GWEIS). Despite recent scientific progresses, challenges such as inadequate study methodology (e.g., correlational studies) and lack of diversity within study samples persist. Recent discoveries of several genetic variants of diseases, could augment and improve, or even challenge, existing understanding of the onset and management of mental illness. Leveraging real-world data (RWD), including electronic health record data (EHRs) focused on social determinant of health alongside biobank data, offers further opportunities to enhance the understanding of gene-environment interactions and inform efforts for reducing disparities in mental healthcare. Increased knowledge can support timely, holistic, evidence-based, and personalized care. Addressing ELSI considerations and maximizing the use of RWD is essential for advancing ethical and inclusive psychiatric genetics research, ultimately improving patient outcomes and promoting equitable access to evidence-based treatments.

## Introduction

To this day, the etiology of mental illnesses remains unclear, and optimizing health outcomes remains difficult across diverse patient populations (Strauss et al., 2019). These challenges arise from the complex interplay of genetic predispositions, environmental influences, and sociocultural factors that characterize psychiatric conditions. Recent advancements in biomedical research offer promising avenues for understanding and addressing the multifaceted nature of mental illnesses. By unraveling the intricate interplay of genetic predispositions, environmental influences, and sociocultural factors, researchers can pave the way for more effective personalized treatment approaches in psychiatry. However, there are several unique ethical, legal, and social (ELSI) hurdles that must be addressed; these include issues such as safeguarding privacy and confidentiality in genetic testing, leveraging all available data sources to improve the representation of diverse populations in research, promoting equitable access to diagnostic tests and treatments, safeguarding against the misuse of genetic data, combating the stigma associated with mental health conditions, and fostering cultural awareness and humility in the development and implementation of assessment tools and treatment strategies (Loughman and Haslam, 2018; Brown et al., 2022; Matalon et al., 2023; Royce et al., 2023).

Innovations in biomedical research, particularly within the clinical trial enterprise, continue to generate evidence that has the propensity to quantify the impact of both intrinsic (i.e., genetics, human anatomy, and physiology, etc.) and extrinsic factors (i.e., dietary preference and habits, personal choices, environment, culture, social determinants of health [SDoH], legal regimes, climate, etc.) on health, wellness, disease development and progression, and treatment outcomes. Indeed, theoretical frameworks have emerged acknowledging these phenomena, such as gene-by-environment (GxE) interaction theories, biopsychosocial models, and social epigenetics, and thus carry important implications for personalized medicine implementation within the field of psychiatry (Loughman and Haslam, 2018; Strauss et al., 2019; Matalon et al., 2023; Royce et al., 2023). Despite these advancements, the clinical management of mental illnesses remains riddled with ongoing ELSI challenges, including the lack of systematic and structured SDoH data collection within mental healthcare settings, inadequate medical-legal and personal support structures for patients, and disparities in accessing high-quality mental healthcare services and innovative clinical trials based on patient geography, financial status, and insurance coverage.

Scientific limitations further exacerbate these challenges, with studies exploring genetic relationships in mental illness often constrained by correlative and multifactorial designs. Additionally, nascent data resources for studying replicable GxE interactions and limitations in studies focused on social epigenetics underscore the need for more comprehensive research approaches. Last but not least, studies focused on social epigenetics, or the impact of SDoH on DNA methylation, are accompanied by multiple limitations, such as limited racial/ethnic/geographic diversity and high dependence on convenience samples (Evans et al., 2021).

Currently, abundant real-world data (RWD) from electronic medical records (EHRs) and other sources exist, creating new opportunities to systematically and prospectively collect phenotype data alongside the discovery of genetic variants of diseases (Bianchi et al., 2024; The All of Us Research Program Genomics Investigators, 2024). Thus, we have arrived at a new frontier to improve our current understanding of how both intrinsic and extrinsic factors contribute to the onset of mental illness and subsequent treatment outcomes. Here we discuss recent evidence from genome-wide association studies (GWAS), phenome-wide association studies (PheWAS), and genome-wide environment interaction studies (GWEIS) to describe this evolving landscape and highlight important ELSI considerations moving forward.

## Emerging GWAS, PheWAS, GWEIS study evidence

Evidence from GWAS, PheWAS, and GWEIS have produced signs linking specific genes to serious mental illness; however, many of these studies have been accompanied by limitations (i.e., poor study methodology, inadequately powered studies, and poor reproducibility) (Hewitt, 2012; Maglione et al., 2018; Mascheretti et al., 2018; Niitsu et al., 2019). Adding to the complexity of replicating these studies, emerging research suggests that stress derived from living within and navigating complex and/or hazardous environments can lead to epigenetic changes (i.e., DNA methylation, histone modification, and non-coding RNA) that may influence the expression of genes that are implicated in psychiatric symptoms, conditions, and disorders (Moore and Zelazo, 2013; Bakusic et al., 2017; Lemche, 2018). Also, GWAS studies to date are largely comprised of individuals of European descent, thus contributing further to a general lack of understanding of the effect of GxE interactions and psychiatric illness in individuals with geographic ancestries outside of Europe (Martin et al., 2017). Real-world studies that account for complex GxE interactions and ancestral diversity can strengthen the current body of evidence from GWAS, PheWAS, and GWEIS studies (The All of Us Research Program Investigators et al., 2019; Levey et al., 2021; Levey et al., 2023).

For instance, a recent study conducted by the Million Veteran Program, iPSYCH2, and Mass General Brigham (MGB) BioBank in the US assessed a sample of 1,054,365 individuals (European n = 886,025; African n = 123,208; admixed American n = 38,289; and East Asian n = 6,843) (Levey et al., 2023). The study found statistically significant single nucleotide polymorphism-based heritability for cannabis use disorder across various genes, such as *SLC36A2*, in every ancestral group excluding East Asians (Levey et al., 2023). This finding has significant implications for mental healthcare systems, serving populations of individuals with a known or unknown risk of cannabis use disorders, particularly in environments with a prevalent social culture of cannabis use, and limited testing for cannabis use disorder (Risi et al., 2020; Sagar et al., 2021; Gorelick, 2023). Relatedly, the U.S. Drug Enforcement Administration has recently proposed a significant revision to cannabis regulation, advocating for its reclassification from a “Schedule I” drug—characterized by lacking accepted medical use and having high potential for abuse—to a less restrictive “Schedule III” status (Bodamer, 2023; Department of Justice DEA, 2024). If it is successful, this shift in federal classification will expand access for consumers and streamline pathways for expanding research on cannabis use disorder and associated health risks (e.g., cannabinoid hyperemesis syndrome and psychosis) (Bartolone, 2017; Hasan et al., 2020).

Lastly, a recent PheWAS study conducted within the PsycheMERGE Consortium (www.psychemerge.com) evaluated polygenic risk scores (PRS) for schizophrenia for association with 1,359 disease categories, including schizophrenia and psychosis, in over 100,000 patients across four large healthcare systems within the United States (US; Geisinger Health System, Mount Sinai Health System, Partners Healthcare System, and Vanderbilt University Medical Center) (Zheutlin et al., 2019). The study found that PRS was strongly associated with schizophrenia, with patients in the highest risk decile of the PRS distribution having over four-fold greater odds of schizophrenia phenotype. This finding further underscores why the complex interplay of interactions between environmental factors and polygenic risk factors should continue to be explored in the etiology of serious mental illness. Although PRS alone may not capture the full complexity of GxE interactions, such evidence of a significant association between schizophrenia’s PRS and disease susceptibility (e.g., substance use disorder) underscores the importance of considering genetic predisposition in the development of schizophrenia in certain environments. However, this PheWAS study focused exclusively on individuals of European ancestry, which likely limits its generalizability to non-ancestral European populations. The challenges of recruiting non-European populations or the lack of diversity in genomic research are well documented (Gerhard et al., 2018; Fatumo et al., 2022; Ju et al., 2022). Such consideration is important given that research and care for serious mental illness, including schizophrenia, can be biased and/or stigmatizing for many individuals of non-European descent (Eylem et al., 2020).

While the aforementioned studies provide valuable insights into psychiatric genetics, they also draw attention to the unique ELSI considerations associated with psychiatric genetics research studies. Firstly, the equitable representation of historically underrepresented groups (e.g., ethnic and racial minority individuals) in clinical research continues to be a persistent challenge (Sharma and Palaniappan, 2021). Nevertheless, findings such as the disparities uncovered in the prevalence of genetic markers for cannabis use disorder among different ethnicities underscore the imperative for meaningful progress in improving diverse representation in clinical research and the need for culturally responsive approaches to assessment and care.

Secondly, it is essential to encompass a deeper examination of how factors like societal norms, cultural practices, and historical contexts intersect with genetic predispositions to shape the development and course of various mental health conditions. Although genetic factors may play a significant role in predisposing individuals to certain conditions, extant research suggests that they represent just one component of the complex interplay between biology, environment, and psychosocial factors (Brown et al., 2022). The etiology and progression of mental illnesses are inherently dynamic, often influenced or exacerbated by environmental stressors, trauma, the strength of an individual’s social support network, and the adequacy of their coping skills. Relying on genetic data in isolation creates a significant risk of oversimplifying the nuanced nature of mental illness and overlooking the diverse factors that significantly contribute to risk, onset, and prognosis.

The implications of study findings should also be considered within the context of the continued disparities in access to mental healthcare and the subsequent uneven distribution of benefits from biomedical advancements across the United States. These disparities are sustained by systemic barriers related to racism/discrimination, socioeconomic status, geographic location, and institutional biases. These persistent disparities indicate that greater efforts are still needed to address barriers to care, ensuring that all individuals, regardless of background or circumstance, have equitable access to evidence-based treatments. By considering the ELSI implications of research design (e.g., ensuring adequate representation of underserved populations in study samples) and findings (e.g., ensuring findings adequately reflect the limitations of genetic determinism), the scientific community can forge a path towards more inclusive and socially responsible research practices. This involves embracing practices that not only aim to advance scientific knowledge but also strive to foster a deeper understanding of the lived experiences of individuals affected by mental illness, accounting for the broader societal implications of biomedical innovation in the mental health field. Adopting this approach is essential for creating a future where mental healthcare is not only effective but also equitable, ethical, and compassionate.

## Considerations for the use and combination of EHR and biobank data

Understanding social and environmental risk factors about a patient to the greatest extent possible is beneficial to mental healthcare providers and critical to GWAS, PheWAS, and GWEIS focused on or guided by social epigenetics. EHRs offer a wealth of information in various formats, including diagnosis codes (International Classification of Diseases, 10th Revision, Clinical Modification [ICD-10-CM]), visit summaries, clinician notes, clinical imaging data, lab results, and/or other patient-level documents. Genetic data and social and economic factors can be captured in many formats, including but not limited to documenting ICD-10-CM codes Z14 and Z15 on genetic factors and Z55-65 Z codes on SDoH factors (see Supplementary Table S1) (Gerhard et al., 2018; Compton and Shim, 2015; Dolin et al., 2023; Robeznieks, 2022).

Recent studies show SDoH Z code documentation is presently low, particularly in mental health settings (Bensken et al., 2022; Heidari et al., 2023; Hendricks-Sturrup et al., 2024). Although, the impetus to conduct GWEIS studies as one mechanism to better understand, predict, and address mental illness, based on emerging evidence, could inspire stronger SDoH Z code and other related or structured RWD (e.g., patient-generated health and laboratory data) documentation across multiple types of health institutions (Bensken et al., 2022; Heidari et al., 2023; Hendricks-Sturrup et al., 2024). Equally urgent is the imperative to guarantee informed consent processes that empower participants to autonomously decide on their involvement in research, free from coercion or undue influence.

In addition to opportunities in leveraging EHR SDoH Z code and other clinical data to enhance the understanding of psychiatric conditions, there is also an ethical imperative to maximize the use of such data in mental health research and clinical practice. By harnessing the vast amount of data stored generally within EHRs, researchers and healthcare providers can ensure that available resources are used appropriately to improve patient outcomes and advance scientific knowledge. Indeed, ethical considerations dictate that we strive to optimize the use of available data sources, including RWD, recognizing its potential to address longstanding health disparities, enhance treatment efficacy, and promote equity in the research evidence base and care delivery. Furthermore, leveraging RWD to its fullest extent, upholds the clinical principles of beneficence and non-maleficence, ensuring that patients receive the highest standard of care while minimizing potential harm associated with ineffective, delayed, or incorrect treatments. Therefore, in addition to the scientific and clinical imperatives, there is also a moral obligation to maximize the use of RWD in psychiatric research and practice.

Mental health and certain SDoH data, such as domestic abuse or violence and/or substance use disorder, are inherently sensitive. Therefore, researchers seeking to leverage SDoH Z codes in combination with other forms of mental health RWD and genomic data should take precautions to protect data subjects from possible stigmatization, discrimination, and other potential repercussions associated with information disclosure. This could include implementing privacy-protective legal mechanisms for research participants or data subjects, such as Certificates of Confidentiality (Social Determinant of Health SDH ICD-10 Z Codes, 2017; samhsa, 2014). Equally urgent is the imperative to guarantee informed consent processes that empower participants to autonomously decide on their involvement in research, free from coercion or undue influence.

Mechanisms to combine SDoH Z code and other EHR data with biobank data could be valuable to elucidate how and/or whether genetic and environmental factors influence mental health over time, and data infrastructure needed to accomplish this is under development. For example, the UK Biobank and All of Us Research Program each hold the capacity to combine genetic data with EHRs to examine factors affecting mental health, aiding in the discovery of genes and genetic variants associated with common psychiatric disorders (Wright et al., 2002; All of Us Research Program, 2024). These biobanks integrate genomic data with RWD curated using the Observational Medical Outcomes Partnership (OMOP) Common Data Model common data model (Collins, 2012; Gaziano et al., 2016; Papez et al., 2023; Data Methods, 2024). Next steps in this development would be to further explore and refine data harmonization strategies given the heterogenous nature of EHR data and data documentation across all, including mental, healthcare institutions. This is especially important given reported challenges in transforming certain types of RWD to fit into the OMOP common data model, the often-subjective (versus objective) nature of reporting mental health RWD, and that OMOP is one of potentially several useful harmonization strategies to ensure consistency, comparability, and reproducibility across diverse datasets (Martone et al., 2018).

## Discussion

Here, we emphasize the need to implement a multifactorial approach to psychiatric research that considers both genetic and environmental influences in the real world. Indeed, there is an intricate interplay between genetic and environmental factors in mental illness underscores the inherent complexities within psychiatry research and practice domains. Today, there is significant potential for RWD to complement or drive innovative genetic studies that might engender a more comprehensive understanding of the genetic underpinnings of psychiatric illness.

While we acknowledge there are limitations of correlational studies and the use of RWD in psychiatric genetics research, it is important to recognize that RWD can provide valuable insights to advance our understanding of complex GxE interactions and better inform treatment strategies. The fact that many studies exploring relationships between genetics and the onset of psychiatric illness are typically correlative rather than causal does not necessarily negate their significance or validity. Instead, it reflects the nature of genetic association studies, such as GWAS, which aim to identify genetic variants associated with disease susceptibility. Indeed, correlational studies grounded in RWD play a crucial role in generating hypotheses and identifying potential genetic factors contributing to psychiatric illness. While they may not establish causation, the valuable insights into the molecular underpinnings of diseases serve as a foundational step toward uncovering causal mechanisms.

GWAS, PheWAS, and GWEIS supported with RWD and with a focus on social epigenetics and other important areas of genetics could strengthen current and future insights into relationships between the environment and the likelihood of observing symptoms of psychiatric disorders. Policy aims to explore the etiological underpinnings of diseases serve as a foundational step toward uncovering causal mechanisms.

GWAS, PheWAS, and GWEIS supported with RWD and with a focus on social epigenetics and other important areas of genetics could strengthen current and future insights into relationships between the environment and the likelihood of observing symptoms of psychiatric disorders. Policy and enterprise initiatives aimed at addressing the global health burden of mental illness should collaborate with patients and families, clinicians, and scientists to accomplish this goal.
