# Supplementary material for: Applying an ELSI lens to real-world data and novel genomic insights for personalized mental healthcare
Source: Front Genet. 2024 Aug 14;15:1444084. doi: 10.3389/fgene.2024.1444084 (PMC11349570; doi:10.3389/fgene.2024.1444084)
Supplement: Supplementary file 1 [file Table1.DOCX]

Supplement

List of International Classification of Diseases, Tenth Revision, Clinical Modification Z Codes conveying genetic or social determinant(s) of health (SDoH) status

| **Genetic or SDoH Z Code** | **Z Code Description** |
| --- | --- |
| Z14 | Genetic carrier |
| Z15 | Genetic susceptibility to disease |
| Z55 | Problems related to education and literacy |
| Z56 | Problems related to employment and  unemployment |
| Z57 | Occupational exposure to risk factors |
| Z58 | Problems related to physical environment |
| Z59 | Problems related to housing and economic circumstances |
| Z60 | Problems related to social environment |
| Z61 | Problems related to negative life events in childhood. |
| Z62 | Problems related to upbringing |
| Z63 | Other problems related to primary support group, including family circumstances |
| Z64 | Problems related to certain psychosocial circumstances |
| Z65 | Problems related to other psychosocial circumstances |
